# Assessing Cognition in CKD Using the National Institutes of Health Toolbox

**DOI:** 10.34067/KID.0000000000000440

**Published:** 2024-04-03

**Authors:** Alexander Zhang, Seth Furgeson, Allison Shapiro, Petter Bjornstad, Zhiying You, Kalie L. Tommerdahl, Angelina Dixon, Erin Stenson, Ester Oh, Jessica Kendrick

**Affiliations:** 1 Division of Renal Diseases and Hypertension, University of Colorado Anschutz Medical Campus, Aurora, Colorado; 2 Section of Pediatric Endocrinology, Department of Pediatrics, Children's Hospital Colorado, University of Colorado Anschutz Medical Campus, Aurora, Colorado; 3 Section of Pediatric Critical Care Medicine, Department of Pediatrics, Children's Hospital Colorado, University of Colorado Anschutz Medical Campus, Aurora, Colorado

**Keywords:** cognition

## Abstract

**Key Points:**

- Participants with CKD had detectable cognitive deficits in fluid cognition, dexterity, and total cognition.
- Sex differences in cognition exist in people with CKD.

**Background:**

CKD is largely an age-related clinical disorder with accelerated cognitive and cardiovascular aging. Cognitive impairment is a well-documented occurrence in midlife and older adults with CKD and affects multiple domains. We examined cognition function and potential sex differences in cognition in adults with CKD.

**Methods:**

We included 105 individuals (49.5% women) with CKD stage 3b–4 (eGFR, 15–44 ml/min) from the Bicarbonate Administration in CKD Trial (NCT02915601). We measured cognitive function using the National Institutes of Health Toolbox Cognition Battery, which assesses cognitive and motor measures, such as executive function, attention, memory, and dexterity. All study measures were collected and analyzed at the study baseline.

**Results:**

The mean (SD) age and eGFR were 61±12 years and 34.9±9.8 ml/min per 1.73 m^2^. Overall, when compared with the National Institutes of Health Toolbox reference population, participants scored, on average, below the 50th percentile across all cognitive domain tests and the dexterity test. Total cognition scores were also below the 50th percentile. Participants with stage 4 CKD had significantly lower fluid cognition scores compared with those with CKD stage 3b (*β*-estimate −5.4 [95% confidence interval, −9.8 to −0.9]; *P* = 0.03). Female participants with CKD performed significantly better on the episodic memory tests and dexterity tests (dominant and nondominant pegboard tests) and had higher crystallized cognition scores, on average, compared with male participants.

**Conclusions:**

Participants with CKD had detectable cognitive deficits in fluid cognition, dexterity, and total cognition. In addition, sex differences in cognitive measures were found among people with CKD.

## Visual Abstract

**Figure:**
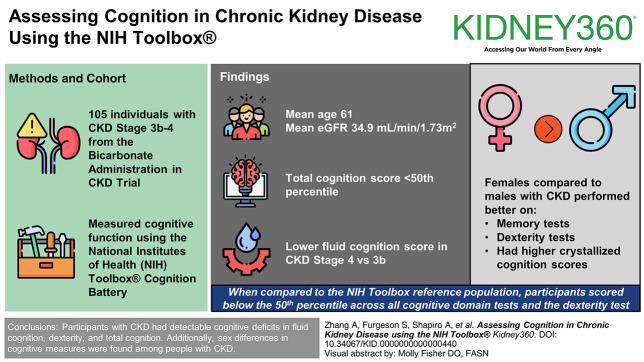

## Introduction

CKD affects nearly 37 million people (15%) in the United States, with individuals older than 65 years, women, and non-Hispanic Black adults being the largest demographic groups affected.^1^ CKD is associated with significant morbidity and mortality.^1^ In particular, cardiovascular disease is highly prevalent in patients with CKD, and greater severity of CKD increases cardiovascular risk.^2^ Furthermore, cognitive dysfunction is also common in patients with CKD, with individuals of all stages of CKD exhibiting higher risk of cognitive dysfunction.^3^ Specifically, the presence and severity of CKD is associated with poorer attention and cognitive control.^4,5^ Alterations in brain structures that contribute to cognitive functions are also evident in patients with CKD.^6^ However, the mechanisms underlying the association between CKD and cognitive dysfunction remain incompletely understood, but likely include shared risk factors, such as cardiovascular disease and CKD-specific risk factors, such as uremic toxins.^6,7^ Subgroups among people with CKD could also be at greater risk of cognitive dysfunction, although few studies have investigated risk stratification by sex or other cofactors.

Importantly, the general population demonstrates sexual dimorphism of cognitive dysfunction and decline.^8^ However, it is unclear whether there are sex differences in cognitive dysfunction among patients with CKD. One meta-analysis failed to find any sex differences; however, numerous cognitive test batteries were represented by the results, making conclusions about the consistency of sex effects across cognitive domains difficult.^9^ Currently, there are limited data comparing cognitive function in men and women with CKD. Accordingly, the objective of this study was to evaluate cognitive function and potential sex differences in people with moderately advanced CKD stages 3b–4.

## Methods

### Participants

We included 105 individuals with CKD stage 3b–4 (eGFR, 15–44 ml/min per 1.73 m^2^) who participated in the National Institutes of Health (NIH)–sponsored Bicarbonate Administration in CKD trial (NCT02915601).^10^ Briefly, inclusion criteria for the trial were age >18 years, CKD stage 3b–4 (eGFR, 15–44 ml/min per 1.73 m^2^), serum bicarbonate 22–27 mEq/L, BP <140/90 mm Hg, and stable antihypertensive, antihyperglycemic, and lipid lowering medications for at least 1 month. All participants were recruited from the Denver metro area, and all study visits took place at the University of Colorado Anschutz Medical Campus. Participants from the parent study were consented to complete optional cognitive assessments at the baseline visit.

### Cognitive Testing

Cognitive testing was performed at the baseline study visit before treatment intervention. All cognitive testing was completed with the NIH Toolbox Cognition Battery (NIHTB-CB), which assesses cognitive, sensory, emotional, and motor functions. The NIHTB-CB tests both fluid (global capacity to reason, ability to learn new things) and crystallized (prior learning and past experiences, based on facts) cognition. Total cognition composite score is based on the average of the Fluid and Crystallized composite score. The NIHTB-CB tests executive functions (Flanker Inhibitory Control and Attention Test and Dimensional Change Card Sort Test), crystallized knowledge (Picture Vocabulary and Oral Reading Tests), working memory (List Sorting), episodic memory (Picture Sequence Memory Test), and cognitive processing speed (Pattern Comparison).^11^ The NIHTB-CB yields individual test scores and composite scores. Fluid composite score includes Flanker Inhibitory Control and Attention Test and Dimensional Change Card Sort Test, Picture Sequence Memory Test, List Sorting working Memory, and Pattern Comparison Tests. The crystallized composite score includes picture vocabulary and oral reading recognition tests. In this study, participants also completed one motor domain to measure motor function (the ability to physically perform tasks). Participants completed a motor dexterity measure, the nine-hole pegboard test. This test records the time required to accurately place and remove nine pegs into a pegboard. Both the dominant and nondominant hands were assessed. The NIHTB-CB was administered by trained study personnel in English or Spanish, as needed. All cognitive measures were performed in the morning. The NIHTB-CB and dexterity test took about 45 minutes to complete.

Fully corrected scores for each cognitive domain test (*e.g*., Flanker test), the composite fluid and crystallized cognition (CC) scores, and the total cognition score were used as the primary outcome variables in this study. Fully corrected scores adjust for age, sex, education level, and race and ethnicity and have a mean of 50 and SD of 10 after correction. A score <50 indicates that the participant is performing below average compared with the referent population. Percentiles of scores for each cognitive test were also determined based on the NIHTB-CB provided cognitive score data for the general referent population.

### Statistical Analyses

Mean and SD were calculated for a continuous variable, and frequency and proportion were provided for a categorical variable. Outcome variables were compared between the male and female participants by using the two-sample *t* test. The *t* test was also performed to compare the outcome variables between those with eGFR ≥30 ml/min per 1.73 m^2^ versus those with eGFR <30 ml/min per 1.73 m^2^. Finally, analysis with a linear regression model was performed to assess the association of each outcome variable with a predictor, both without and with adjustment for a covariate. Complete case analysis has been performed, and the two-sided significance level of 0.05 was used to make a conclusion. All analyses were performed by using SAS version 9.4 (SAS Institute Inc., Cary, NC).

## Results

Baseline characteristics of the study participants are shown in Table 1. Participants were on average aged 61.6 (SD, 11.7) years, 49.5% were female, and were predominantly non-Hispanic White (77.4%). Participants had advanced kidney disease with a mean (SD) eGFR of 35 (10) ml/min per 1.73 m^2^, and 93% had a history of hypertension.

**Table 1:** Baseline participant characteristics

Characteristic	Participants (*n*=105)
Age, yr	61.6±11.7
Female, *No.* (%)	52 (49.5)
**Race, *No.* (%)**	
White	82 (78.1)
Black	13 (12.4)
**Ethnicity, *No.* (%)**	
Hispanic	24 (22.9)
Diabetes, *No.* (%)	48 (45.7)
Hypertension, *No.* (%)	98 (93.3)
**Etiology of kidney disease, *No.* (%)**	
Diabetes	46 (43.8)
Hypertension	21 (20.0)
PKD	5 (4.8)
GN	13 (12.3)
Drugs/toxins	8 (7.6)
CHF, *No.* (%)	8 (7.6)
CAD, *No.* (%)	12 (11.4)
History of stroke, *No.* (%)	7 (6.7)
**Smoking status, *No.* (%)**	
Current	8 (7.6)
Former	34 (32.4)
Use of ACEi/ARB, *No.* (%)	69 (66.3)
Use of *β*-blocker, *No.* (%)	46 (43.8)
Use of aspirin or antiplatelet medication, *No.* (%)	43 (41.0)
Body mass index, kg/m^2^	30.0±5.9
Systolic BP, mm Hg	120.1±14.9
eGFR, ml/min per 1.73 m^2^	34.9±9.7
Urine ACR, mg/g, median (IQR)	86.4 (17.1–384.4)

All values are mean (SD) unless otherwise specified. ACEi, angiotensin converting enzyme inhibitor; ACR, albumin-creatinine ratio; ARB, angiotensin receptor blocker; CAD, coronary artery disease; CHF, congestive heart failure; PKD, polycystic kidney disease.

Overall, when compared with the NIH Toolbox reference population, participants scored, on average, below the 50th percentile across all cognitive domain tests and the dexterity test (Table 2). Total cognition scores were also below the 50th percentile, on average, in the current CKD population. Participants scored in the lowest percentiles, on average, on the Flanker test, which examines attention and inhibitory control and on the pegboard test which measures dexterity. When examining the proportion of patients with significantly impaired cognition (scores below the 25th and 16th percentile), we found that more than 50% had executive function (Flanker test) scores below the 25th percentile and 29.7% had scores below the 16th percentile (Table 3). Forty five percent of the entire population had fluid cognition (FC) scores below the 25th percentile and 40.7% had scores below the 16th percentile (Table 3). Nearly 32% of patients had total cognition scores below the 25th percentile. More than half of the patients had dominant dexterity scores less than the 25th percentile (Table 3).

**Table 2:** Cognition scores and dexterity scores for all participants

Variable	Fully Corrected Score	Percentile
**FC measures**		
Executive function		
*Flanker test*	42.5±7.7	28.4±21.2
*Card sort test*	50.5±10.9	48.4±31.8
Working memory		
*List sort*	48.8±10.2	46.1±29.9
Episodic memory		
*Picture sequence*	48.4±9.4	43.3±28.5
Cognitive processing speed		
*Pattern comparison*	44.4±11.9	35.9±30.9
**Total cognition measures**		
FC	45.2±10.4	38.1±30.4
CC	51.7±8.7	54.1±27.6
Total cognition (FC+CC)	48.2±9.0	44.7±29.1
**Motor domain**		
Dexterity		
*Pegboard dominant hand*	42.2±10.1	27.6±24.0
*Pegboard nondominant hand*	43.7±8.7	31.8±24.2

All values are mean±SD. CC, crystallized cognition; FC, fluid cognition.

**Table 3:** Proportion of CKD stage 3b and stage 4 with significantly impaired cognition (scores below the 25th percentile and 16th percentile)

Variable	All	CKD Stage 3	CKD Stage 4	*P* Value
**Scores below the 25th percentile**				
Executive function, *No.* (%)				
*Flanker test*	48 (52.7)	28 (54.9)	20 (50.0)	0.05
*Card sort test*	26 (28.6)	11 (21.6)	15 (37.5)	0.37
Working memory, *No.* (%)				
*List sort*	27 (29.7)	9 (17.6)	18 (45.0)	0.99
Episodic memory, *No.* (%)				
*Picture sequence*	34 (37.4)	16 (31.4)	18 (45.0)	0.32
Cognitive processing speed, *No.* (%)				
*Pattern comparison*	44 (48.4)	22 (43.1)	22 (55.0)	0.28
FC, *No.* (%)	41 (45.1)	19 (37.3)	22 (55.0)	0.61
CC, *No.* (%)	15 (16.5)	8 (15.7)	7 (17.5)	0.32
Total cognition, *No.* (%)	29 (31.9)	13 (25.5)	16 (40.0)	0.48
Dexterity, *No.* (%)				
*Pegboard dominant hand*	49 (53.8)	26 (51.0)	23 (57.5)	0.22
*Pegboard nondominant hand*	42 (46.2)	20 (39.2)	22 (55.0)	0.26
**Scores below the 16th percentile**				
Executive function, *No.* (%)				
*Flanker test*	27 (29.7)	13 (25.5)	14 (35.0)	0.29
*Card sort test*	21 (23.1)	10 (19.6)	11 (27.5)	0.95
Working memory, *No.* (%)				
*List sort*	17 (18.7)	6 (11.8)	11 (27.5)	0.64
Episodic memory, *No.* (%)				
*Picture sequence*	23 (25.3)	12 (23.5)	11 (27.5)	0.92
Cognitive processing speed, *No.* (%)				
*Pattern comparison*	36 (39.6)	17 (42.5)	19 (37.3)	0.71
FC, *No.* (%)	37 (40.7)	17 (33.3)	20 (50.0)	0.25
CC, *No.* (%)	9 (9.9)	4 (7.8)	5 (12.5)	0.39
Total cognition, *No.* (%)	25 (27.5)	12 (23.5)	13 (32.5)	0.92
Dexterity, *No.* (%)				
*Pegboard dominant hand*	40 (44.0)	19 (37.3)	21 (52.5)	0.94
*Pegboard nondominant hand*	34 (37.4)	17 (33.3)	17 (42.5)	0.34

CC, crystallized cognition; FC, fluid cognition.

We also examined differences in cognitive scores in participants with CKD stage 4 compared with stage 3 (Table 4). Participants with more advanced CKD (stage 4) had significantly lower FC scores compared with those with CKD stage 3b (*β*-Estimate −5.4 [95% confidence interval, −9.8 to −0.9], *P* = 0.03). Participants with CKD stage 4 also had significantly lower scores for working memory (List Sorting test *β*-estimate: −6.2 [95% confidence interval, −10.3 to −2.0], *P* = 0.005) compared with participants with stage 3b. There were no significant differences in CC, total cognition, or dexterity scores between participants with CKD stage 3b or 4. We also examined the proportion of participants with significantly impaired cognition (scores below the 25th and 16th percentiles) by CKD stage (Table 3). More than 50% of patients with CKD stage 3b and 4 had executive function test (Flanker test) scores below the 25th percentile. More patients with CKD stage 4 had working memory scores, cognitive processing speed scores, episodic memory scores, and total cognition scores below the 25th and 16th percentiles than CKD stage 3b, but the differences did not reach statistical significance.

**Table 4:** Difference in cognition scores in participants with CKD stage 4 compared with stage 3b

Variable	CKD Stage 3b		CKD Stage 4		*P* Value for Percentile
	Fully Corrected Score	Percentile	Fully Corrected Score	Percentile	
**FC measures**					
Executive function					
*Flanker test*	42.8±7.4	28.4±20.0	42.7±7.3	27.6±22.0	0.86
*Card sort test*	52.4±10.4	52.7±31.0	48.3±10.7	41.2±29.7	0.08
Working memory					
*List sort*	52.1±9.5	55.9±28.0	45.9±10.0	37.5±29.3	0.004^a^
Episodic memory					
*Picture sequence*	50.9±9.1	50.7±29.5	47.1±9.3	39.1±25.4	0.06
Cognitive processing speed					
*Pattern comparison*	44.9±13.1	38.1±32.5	42.9±10.1	29.8±26.2	0.20
**Total cognition measures**					
FC	48.1±10.1	45.4±31.3	42.7±10.0	30.6±27.8	0.03^a^
CC	52.7±7.3	58.4±25.4	53.1±9.7	55.3±28.2	0.59
Total cognition (FC+CC)	50.5±8.1	51.2±28.6	47.6±8.9	40.4±29.3	0.09
**Motor domain**					
Dexterity					
*Pegboard dominant hand*	43.1±9.4	28.7±23.5	40.4±10.2	24.4±21.9	0.39
*Pegboard nondominant hand*	44.9±9.7	35.7±27.6	42.3±6.6	26.4±17.9	0.08

All values are mean±SD. CC, crystallized cognition; FC, fluid cognition.

a Fully corrected scores *P* value < 0.05.

We tested for correlations between eGFR and cognitive function. There was a significant negative correlation between eGFR and CC (−0.28, *P* = 0.004). eGFR was not significantly correlated with any other cognitive measure or with dexterity. We did not find any significant correlation between serum bicarbonate and cognitive function or dexterity.

When examining cognition by sex, female participants scored in a significantly lower percentile than male participants on the Flanker test, *P* = 0.006 (Table 5). Female participants with CKD performed significantly better on the picture sequence test and the dominant and nondominant pegboard tests and had higher CC scores, on average, compared with male participants (Table 5). There were no significant differences in fluid or total cognition scores between male and female participants with CKD.

**Table 5:** Cognitive scores across sex

Variable	Men		Women		*P* Value for Percentile
	Fully Corrected Score	Percentile	Fully Corrected Score	Percentile	
**FC measures**					
Executive function					
*Flanker test*	43.2±8.0	34.0±23.7	41.9±7.4	22.7±16.7	0.006
*Card sort test*	51.7±10.5	54.1±32.5	49.3±11.3	42.7±30.2	0.07
Working memory					
*List sort*	47.7±10.0	44.7±29.6	50.0±10.5	47.5±30.3	0.64
Episodic memory					
*Picture sequence*	46.1±8.7	33.2±26.0	50.7±9.5	53.5±27.6	0.001^a^
Cognitive processing speed					
*Pattern comparison*	45.5±12.0	38.8±32.0	43.3±11.8	32.8±29.7	0.32
**Total cognition measures**					
FC	44.9±10.7	39.1±31.0	45.6±10.2	37.1±30.1	0.75
CC	49.7±8.7	50.9±27.8	53.8±8.3	57.4±27.2	0.23^a^
Total cognition (FC+CC)	49.5±9.2	43.3±28.7	47.0±8.7	46.2±29.7	0.62
**Motor domain**					
Dexterity					
*Pegboard dominant hand*	41.5±11.3	20.3±21.4	42.9±8.6	35.5±24.2	0.001
*Pegboard nondominant hand*	41.8±9.5	24.3±22.2	45.8±7.3	40.0±23.9	0.001^a^

All values are mean±SD. CC, crystallized cognition; FC, fluid cognition.

a Fully corrected scores *P* value < 0.05.

## Discussion

Overall, we found that people with CKD had detectable cognitive deficits in FC, dexterity, and total cognition when compared with the general population. Our results are consistent with the existing literature where deficits in multiple cognitive domains are observed among individuals with CKD.^6^ We also found that participants with more advanced CKD (stage 4) had lower cognitive scores than those with earlier stage CKD (stage 3b). Our results add to the existing data reporting that a substantial proportion of people with CKD experience cognitive dysfunction and are at increased risk of dementia.^6,12^

In this study, we found that people with more advanced CKD had lower cognitive scores than those with earlier stage CKD. Our results support findings from a meta-analysis showing that risk of cognitive dysfunction increases with lower eGFR.^13^ A study completed in a pediatric population found that longer duration, rather than severity, of kidney disease correlated with poorer performance on cognitive testing.^14^ In our study, we did not collect data on duration of kidney disease so were unable to assess this; however, further research into this area is necessary. Despite missing data on duration of CKD, our results successfully support the proposition that the management of people with CKD should include regular screening for cognitive dysfunction.

Importantly, we found that dexterity was also impaired in individuals with CKD. Previous studies have also shown reductions in hand dexterity in people with CKD.^15^ Hand dexterity relies on the ability to make coordinated and accurate finger and hand movements.^16^ Hence, dexterity may be reflective of one's ability to perform movements essential for activities of daily living and impairments may negatively affect quality of life and independence. While interventions to improve cognitive function in CKD are less well studied, dexterity has been shown to improve with intradialytic exercise rehabilitation.^17^ Thus, assessing dexterity in people with CKD and detecting decrements in function could help facilitate earlier intervention to improve quality of life and independent living.

Sex differences are well documented in the cognitive function and impairment literature among groups without CKD. For example, meta-analyses examining cognition in Alzheimer disease reveal that men perform better with verbal and visuospatial tasks, as well as episodic and semantic memory, compared with women.^18,19^ Women with Lewy Body dementia more often had poorer cognitive function and more severe neurologic problems compared with men.^18,19^ In people with CKD, sex differences in disease progression, mortality, and CKD-related pathologies have also been well documented.^20^ In our study, we found that female participants scored higher with manual dexterity, processing speed and fluidity, language comprehension, and episodic memory, whereas men scored higher with attention and inhibitory control. There was no difference in total cognition between male and female participants, aligning with a meta-analysis in CKD which also did not find sex differences in cognitive function.^9^ However, it is possible that with a larger study population, we would have found differences in total cognition between male and female participants. Further work is needed to examine how sex may compound the effect of CKD on CKD-related cognitive dysfunction.

The mechanisms behind cognitive dysfunction in CKD are not well understood. However, genetics may contribute, at least in part. In pediatric populations with CKD, cognitive dysfunction may result from a genetic disorder that causes both CKD and cognitive issues.^21^ In adults, genetic factors may play less of a role overall, but any genetic variants that increase the risk of cognitive dysfunction in the general population may also occur in people with CKD and must be considered.^6^ Vascular disease is also common in people with CKD,^22^ and vascular-related damage can lead to reduced cerebral blood flow. Indeed, studies have shown that there is reduced cerebral blood flow and oxygenation in individuals with CKD.^23^ People with CKD also have a higher prevalence of subclinical cerebrovascular disease, as evidenced by more white matter lesions and silent brain infarcts detected on brain imaging.^24^ Both white matter lesions and silent brain infarcts have been associated with an increased risk for cognitive decline, dementia, and stroke in people with CKD.^25^ Finally, uremic toxins may also result in cognitive dysfunction. Of the known uremic toxins, 9% are associated with negative neurological effects.^6^ Further work is needed to determine the mechanisms by which cognitive dysfunction occurs in CKD.

There were several limitations to our study that should be considered when interpreting the results. First, our cohort population was relatively small (*n*=105) and only included participants with CKD stage 3b or 4. In addition, while we found some differences in cognition between stages 3b and 4, we had a smaller number of participants with CKD stage 4 (*n*=40) and thus may have been underpowered to see additional differences. We also did not have data on duration of CKD or family history of cognitive dysfunction. We did not have any data on anemia status of participants as anemia has been associated with cognitive decline in CKD. A strength of our study was that approximately 50% of participants were female, allowing us to examine cognition across sex. Future studies with the NIH Toolbox should expand to include individuals with all stages of CKD, as well as people with ESKD to examine how the rate and severity of cognitive decline changes on the basis of disease severity.

CKD is a disease known to negatively affect cognition as it progresses in severity. The ability to accurately evaluate cognition of individuals with CKD would help providers determine the rate, extent, and prognosis of disease over time, factoring into potential future treatment options. The NIH Toolbox is a newly developed method of assessing multiple important cognitive domains, thus giving providers a better understanding of what regions of the brain may be most affected by CKD pathophysiology. Future studies are needed to elucidate the mechanisms by which cognitive dysfunction occurs in CKD and to identify novel therapies to improve cognitive function in CKD.

## Supplementary Material

## Data Availability

All data are included in the manuscript and/or supporting information.
